# Health Communications Theory-Based Text Message Reminders Boost Special Supplemental Nutrition Program for Women, Infants, and Children (WIC) Appointment Attendance Among American Indian Populations

**DOI:** 10.3390/nu17071112

**Published:** 2025-03-22

**Authors:** Emily M. Melnick, Nicole Vaudrin O’Reilly, Francesco Acciai, Cori Lorts, Mindy Jossefides, Punam Ohri-Vachaspati

**Affiliations:** 1 College of Health Solutions, Arizona State University, Phoenix, AZ 85004, USA; facciai@asu.edu (F.A.); pohrivac@asu.edu (P.O.-V.); 2 Inter Tribal Council of Arizona, Inc., Phoenix, AZ 85004, USA; nicole.vaudrino'reilly@itcaonline.com (N.V.O.); mindy.jossefides@itcaonline.com (M.J.); 3 Independent Researcher, Flagstaff, AZ 86001, USA; corilorts@gmail.com

**Keywords:** WIC program, American Indians, text messaging, technology, health communication

## Abstract

**Background/Objectives:** The Special Supplemental Nutrition Program for Women, Infants, and Children (WIC) improves the health of nutritionally at-risk populations. However, engagement challenges, such as missed appointments and unredeemed food benefits, may limit program efficacy. Barriers to engagement are heightened among American Indian populations, who often experience disproportionately high levels of participation-related challenges. This study assessed whether newly developed health communication theory-based text messages incorporating persuasive language and different message framing (i.e., gain-framed and loss-framed) improved WIC appointment attendance and food benefit redemption rates, above and beyond standard information-based reminders. **Methods:** The sample included participants served by the Inter Tribal Council of Arizona WIC between the months of September 2022 and February 2023 who (a) had an appointment during the intervention period (n = 7584) or (b) were eligible to receive a reminder text about unused food benefits during the intervention period (n = 2177). A three-phase intervention design was used, with each phase lasting six weeks. During the baseline phase, participants received standard information-based text messages, while during the two intervention phases, participants received newly developed messages using (1) gain-framed and (2) loss-framed persuasive language. Difference-in-difference regression analyses compared whether differences in outcomes (i.e., appointment attendance and monthly food benefit redemption rates) between participants who received reminder messages and those who did not differed over intervention phases. **Results:** Receipt of both gain-framed and loss-framed theory-based messages was associated with higher appointment attendance, when compared to receipt of standard information-based messages during baseline (*p* = 0.003 and *p* = 0.01, respectively). Neither the gain-framed nor loss-framed messages were associated with higher food benefit redemption rates than standard messages. **Conclusions:** Results indicated that persuasive communication theory-based text reminders may be an effective, low-cost strategy to boost WIC appointment attendance among American Indians.

## 1. Introduction

The Special Supplemental Nutrition Program for Women, Infants, and Children (WIC) improves the health of nutritionally at-risk women, infants, and children by providing supportive nutrition education for caregivers and healthy foods to eligible households [1]. WIC-participating households receive monthly food benefit packages designed to supplement participants’ diets with age-appropriate nutrient-dense food. For example, infant food packages include items such as infant cereal and baby foods, while child food packages include items such as milk, eggs, and fruits and vegetables [1]. There are many health benefits for those who participate in WIC. Children within households who participate in WIC have healthier diets compared to those within eligible households who do not participate [2,3]. However, attending scheduled WIC appointments can be challenging, especially for families with higher levels of stress and cognitive overload, which is oftentimes experienced by caregivers with infants and young children at higher food insecurity risk [4,5,6]. As a result, participants often miss scheduled appointments and leave monthly food benefits unredeemed [7,8,9].

These challenges may be heightened among American Indian populations, who typically experience disproportionately high poverty and food insecurity rates, which have been even further exacerbated by the COVID-19 pandemic [10,11,12]. A survey conducted by the Native American Agriculture Fund in 2021 showed that during the early months of the pandemic, 59% of respondents with at least one child under age five in their household experienced food insecurity, and 33% experienced very low food security, which is defined by the U.S. Department of Agriculture (USDA) as reporting multiple indications of disrupted eating patterns and reduced food intake [13,14]. Correspondingly, American Indian families with young children often experience higher stress levels than other racial/ethnic groups [15,16,17]. Further, American Indian WIC participants—particularly participants living on tribal lands—redeem relatively less of their monthly food benefits compared to other racial and ethnic groups [18,19], and WIC participation declined by nearly eight percent among Indian Tribal Organization WIC agencies that serve American Indian communities during the first two years of the COVID-19 pandemic, despite concurrently rising food insecurity rates [20]. It is, therefore, important to identify and evaluate strategies that are effective in increasing participant engagement in the WIC program among American Indian populations, who are typically underrepresented in studies of national scope [21].

Many state and local WIC agencies use text message reminders to notify participants about upcoming appointments and the expiration of their monthly food benefits, with the goal of enhancing program engagement [22]. While text message reminders have shown some effectiveness in reducing missed appointment rates, their impact has been modest and examined only in healthcare settings [23,24,25]. Therefore, it is worth considering whether the language used in reminder text messages affects participants’ engagement. Research indicates that incorporating persuasive language grounded in health communication theories improves appointment attendance rates compared to merely informative text message reminders that lack persuasive language [26,27]. Using health communication theories, messages can be framed to emphasize specific pieces of information aimed at increasing their personal relevance to the recipient, thus motivating them to engage in the targeted behavior. For instance, social norm messages can effectively highlight the prevalence of positive behaviors among peers, as individuals often align their actions with perceived social norms [28]. Indeed, Senderey et al. found that text message reminders highlighting social norms significantly reduced the number of missed hospital appointments compared to standard information-based reminders [27].

Messages can also be framed to highlight the positive outcome associated with a behavior (a gain frame) or focus on the negative consequences of not performing the behavior (a loss frame) [29,30]. Gain- and loss-framed messaging has been studied extensively for a variety of nutrition-related topics, including fruit and vegetable consumption [31], calcium intake [32], a healthy diet [33], and sugar-sweetened beverage consumption [34]. The results of this research were mixed; however, gain-framed messages were theorized to be more effective for the general public when knowledge of a subject is low [35,36].

Understanding whether incorporating persuasive theory-based language and message framing into reminder text messages improves WIC program engagement can help to optimize WIC agency communication with participants. Evidence that incorporating theory-based language into messages boosts participant engagement in the WIC program would provide important information to WIC agencies in search of low-cost and easily implementable strategies to improve engagement. This evaluation examines the impacts of reminder text messages developed using health communication theories on program engagement among participants served by the Inter Tribal Council of Arizona, Inc. (ITCA) WIC (Phoenix, AZ, USA), compared to standard, information-based reminder messages that the ITCA WIC had already been sending to participants. The objective of the current intervention evaluation is to assess whether text message reminders incorporating health communication theory-based message framing improves (1) appointment attendance and (2) food benefit redemption rates among ITCA WIC participants, compared to standard reminders. We hypothesize that both appointment attendance and food benefit redemption rates will be higher after receiving theory-based messages, compared to standard information-based reminders.

## 2. Materials and Methods

### 2.1. Setting

This intervention was conducted among participants served by ITCA WIC between the months of September 2022 and February 2023. The ITCA is a non-profit organization whose members are the 21 tribal nations in Arizona. The ITCA serves as a WIC state agency. ITCA WIC’s monthly caseload is approximately 7000 participants. Most ITCA WIC local agencies (12 out of 13 agencies) provide services through clinics and satellite locations on tribal lands.

### 2.2. Text Message Development

To develop new health communication theory-based text messages tailored to the unique population served by ITCA WIC, a comprehensive message development and pretesting process was undertaken. First, a review of the relevant health communication and WIC communication literature was conducted. Next, a health communications expert (C.L.) developed an initial catalog of 16 reminder text messages, outlined in Table 1, that incorporated persuasive gain-framed or loss-framed language based on health communication theories. All food benefits reminder messages highlighted WIC dollars to purchase fruits and vegetables, because a COVID-19-related federal policy increased the amount of the cash value benefit (CVB) to purchase fruits and vegetables available to eligible WIC beneficiaries starting in March 2021, and continuing resolutions extended these increases throughout the study period [37,38]. ITCA WIC state agency staff (M.J. and N.V.) provided ongoing feedback on the catalog of messages to ensure relevancy for the audience and to ensure that the newly developed texts were appropriate for the literacy level of the target population.

After the research team came to a consensus on the reminder messages to be considered in the pretesting phase, the lead author of this paper (E.M.) conducted key informant interviews with ITCA WIC local agency staff to gather feedback on the developed messages. Interviews were conducted with 15 local agency employees, representing 8 ITCA WIC local agencies. To minimize respondent burden, half of the interviewees (randomly selected) were asked to provide feedback on food benefits reminder messages and the breastfeeding-related appointment reminder messages, and half were asked to provide feedback on general appointment and the nutrition education appointment reminder messages. Two-thirds (10/15) of the interviewees had either previously participated or were currently participating in WIC. Interviewees were asked to rate how clear, persuasive, and personally relevant each message was, as well as what they particularly liked or disliked about each message using questions based on previous research [39,40]. Based on this feedback, the research team selected final messages to be delivered to ITCA WIC participants.

Messages that tested particularly well were the ones that incorporated health-focused language. Gain-framed and loss-framed messages tested similarly in interviews. Therefore, all final messages included health-focused language. A two-phase approach evaluated (1) gain-framed messaging with health-focused language (e.g., “You work hard to keep your family healthy—WIC is here to help! WIC saves you money on healthy food”) and (2) loss-framed messaging with health-focused language (e.g., “You work hard to keep your family healthy—WIC is here to help! Don’t miss out on healthy WIC foods”). All newly developed messages incorporating health communication-based framing, as well as previously sent standard information-based messages, are available in Supplementary Materials (Table S1).

### 2.3. Evaluation Procedures

#### 2.3.1. Outcomes

The outcomes of interest in this study included (a) appointment attendance rates and (b) overall food benefit redemption rates. All outcome data came from the ITCA WIC management information system, which allows for the production of monthly reports related to program operations. Monthly appointment attendance reports were generated to measure participant appointment attendance, which was coded as a binary variable: (1) attended appointment or (0) did not attend appointment (including no-show, cancelled, and rescheduled appointments). Monthly benefits issued and redeemed reports were generated to measure household overall food benefits redemption, coded continuously between 0 and 100 percent. CVB redemption by itself—also coded continuously between 0 and 100 percent—was examined in a separate sensitivity analysis because the newly developed reminder texts included specific language about redeeming fruits and vegetables with WIC dollars. Appointment attendance was analyzed at the individual level, whereas redemption outcomes were assessed at the household level because monthly food benefits are issued and redeemed at the household level.

#### 2.3.2. Exposure

The exposure of interest in this intervention study was the successful receipt of reminder text messages. ITCA WIC contracts with the Teletask (Teletask Inc., Fair Oaks, CA, USA) text messaging platform to send text messages to participants who provide valid cell phone numbers. On average, 90% of the ITCA WIC participants provide a valid cell phone number for their household upon enrollment and consent to receive text messages. Messages sent include upcoming appointment reminder texts to all participants one day before their appointments and food benefits reminder texts two weeks before the monthly benefits expiration date to participants who had not yet redeemed any of their monthly household food benefits.

To determine whether participants successfully received reminder text messages, monthly reports, including message receipt status, were generated from the Teletask messaging platform. For each message sent, the platform recorded the type of message sent (e.g., general appointment reminder or benefits reminder), delivery time, and delivery status (e.g., successful delivery or failed delivery). Exposure to each reminder text message based on message receipt status was considered as a binary variable (exposed to text message vs. not exposed).

For appointment attendance outcomes, the non-exposed group included participants who did not receive the text due to (a) previously opting out of receiving text messages, (b) message delivery failure, or (c) no message was sent due to either the participant not providing a valid cell phone number or scheduling the appointment less than two days in advance. For the food benefit redemption outcomes, the non-exposed group included households who did not receive the text due to (a) previously opting out of receiving text messages or (b) message delivery failure.

**Figure 1:**
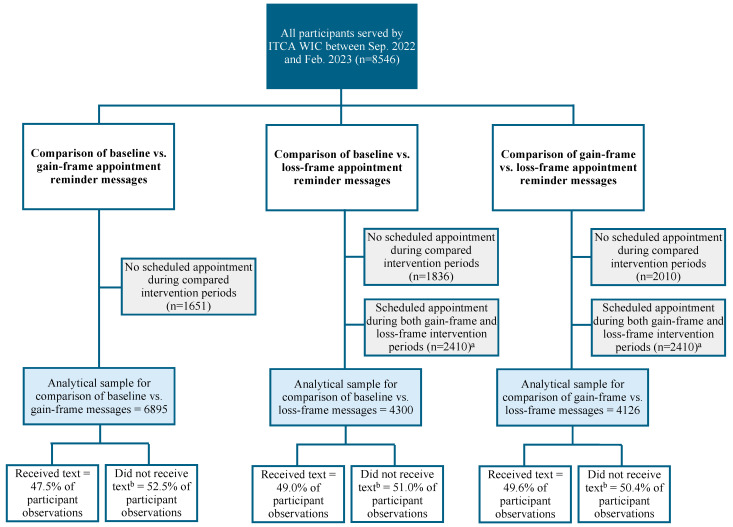
Description of the analytical samples for comparisons assessing impacts of theory-based text messages on appointment attendance rates among Inter Tribal Council of Arizona WIC participants. ^a^ Excluded participants to avoid potential carryover effects from the gain-framed phase. ^b^ Includes participant observations for which recipients did not receive text due to (a) previously opting out of receiving text messages, (b) message delivery failure, (c) not providing a valid cell phone number, or (d) scheduling an appointment less than two days in advance.

**Figure 2:**
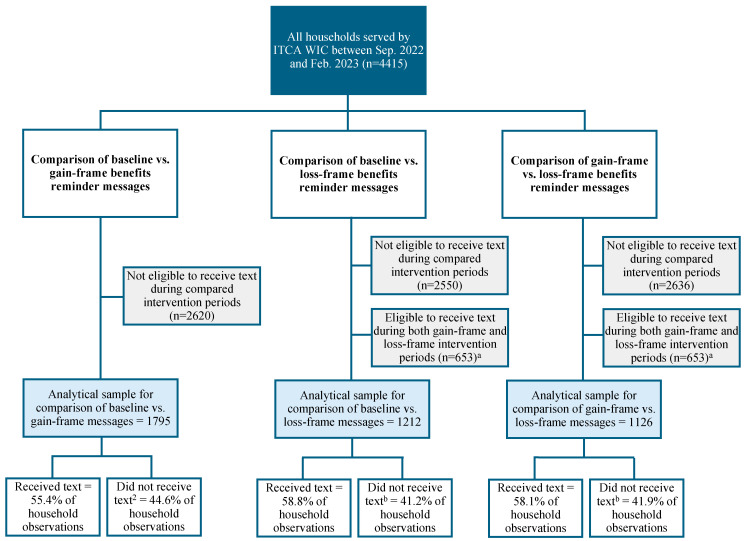
Description of the analytical samples for comparisons assessing impacts of theory-based text messages on household food benefit redemption rates among Inter Tribal Council of Arizona WIC-participating households. ^a^ Excluded households to avoid potential carryover effects from the gain-framed phase. ^b^ Includes household observations for which recipients did not receive text due to (a) previously opting out of receiving text messages, or (b) message delivery failure.

The proportion of participants who did not receive texts (i.e., the non-exposed group) was stable across the intervention period, as shown in Figure 1 and Figure 2. The distributions of reasons for not successfully receiving a text message were also stable across study periods. For example, the proportion of texts not received because participants had previously opted out was comparable during the baseline phase (6%), gain-framed phase (7%), and loss-framed phase (8%). The proportion of texts not received due to failed delivery was also similar during the baseline phase (16%), gain-framed phase (17%), and loss-framed phase (17%).

Of note, it is likely that dissimilarities (e.g., sociodemographic disparities) between participants assigned to the exposed and non-exposed groups at each study phase existed. For example, 62% of households that did not receive a given appointment reminder text across the intervention reported participating in the Supplemental Nutrition Assistance Program (SNAP)—another federal food assistance program with a lower income eligibility threshold than WIC [41]—while 45% of households that did receive a text reported participating in SNAP. Therefore, to ensure that such compositional differences between groups did not affect the analysis, the study design did not compare outcomes across these groups but instead compared differences between exposed and unexposed groups across study phases using a difference-in-difference (DiD) approach. This approach is most suited for these analyses under the assumption that the differences between the exposed and unexposed groups would have remained the same in the absence of the intervention.

#### 2.3.3. Intervention Design

The research team initially explored the possibility of conducting a randomized trial; however, compatibility issues and limited integration between the ITCA WIC management information system and the Teletask platform made that impossible. Therefore, a DiD approach, based on a three-phase intervention design, assessed the impacts of differently framed reminder texts. Using this approach, impacts of text messages were evaluated by comparing whether differences in outcomes for those who were exposed to text messages vs. those who were not exposed differed across intervention phases. The intervention phases included (1) a baseline phase spanning between 15 September and 31 October 2022, during which standard reminder messages were sent, (2) a gain-framed phase spanning between 1 November and 15 December 2022, during which theory-based gain-framed messages were sent, and (3) a loss-framed phase spanning between 1 January and 15 February 2023, during which theory-based loss-framed messages were sent. Text messages sent between 15 December and 1 January were not evaluated, as attendance during the holiday period can be erratic. Test data were obtained for the first week following each study phase transition from Teletask to verify that the correct messages were being sent.

### 2.4. Analytical Sample

The eligible sample included all participants served by ITCA WIC during the intervention period. Analytical samples and inclusion and exclusion criteria for each specific comparison are displayed in Figure 1 and Figure 2 and described for each outcome below.

#### 2.4.1. Appointment Attendance Sample

The analytical sample for each appointment attendance comparison (Figure 1) included participants who had at least one scheduled appointment during at least one of the two compared phases (i.e., baseline vs. gain-framed messages, baseline vs. loss-framed messages, or gain-framed vs. loss-framed). Because the loss-framed phase followed the gain-framed phase, learning effects could not be ruled out. To avoid this, participants who had scheduled appointments during both phases were excluded from the sample for the two comparisons involving the loss-framed message.

#### 2.4.2. Monthly Food Benefits Redemption Sample

The analytical sample for each food benefits redemption comparison (Figure 2) included households who were eligible to receive a food benefits reminder text, i.e., households that had not redeemed any of their benefits two weeks prior to the monthly benefits expiration date during at least one of the two compared phases. As in appointment attendance comparisons, to avoid potential learning effects, households observed during both gain-framed and loss-framed periods were excluded in comparisons involving the loss-framed text messages.

The analytical sample for the CVB redemption outcome (Figure S1 included in Supplementary Materials) explored in a sensitivity analysis was slightly smaller than the sample for the overall food benefits redemption outcome because only households containing a woman and/or child beneficiary received CVBs in their monthly food benefits package.

### 2.5. Covariates

All analytical models included the race (American Indian vs. not) and ethnicity (Hispanic vs. not) of the most senior participating member of the household, household SNAP participation (yes vs. no), the number of WIC-participating infants, children, and women in the household, all coded continuously, as well as the local agency attended by the household (as a nominal variable with 13 categories) as control variables. These variables were captured using monthly client detail reports generated from the ITCA WIC management information system.

### 2.6. Statistical Analysis

Regression models based on a DiD approach evaluated whether the difference in appointment attendance/benefits redemption rates between (a) individuals who successfully received text messages (i.e., the exposed group) and (b) individuals who did not (i.e., the non-exposed group) differed across phases of the intervention period. Specifically, analyses compared differences in outcomes for exposed and non-exposed groups for (a) baseline vs. gain-framed messages, (b) baseline vs. loss-framed messages, and (c) gain-framed vs. loss-framed messages.

The DiD approach is often used to infer causality from observational studies. Its primary strength lies in leveraging within-group variation over time, producing more robust estimates by comparing changes in outcomes before and after the intervention for both the treatment and the comparison groups [42]. By differencing out pre-treatment differences between exposed and non-exposed groups, DiD helps mitigate selection bias and other unobserved heterogeneity issues [43]. DiD can also accommodate various forms of treatment assignment, making it a suitable tool for analyzing a wide range of policy interventions and natural experiments [44].

These characteristics make the DiD approach particularly suitable for the current analysis evaluating newly developed theory-based text messages in the absence of a randomized design. Using DiD, we can estimate the impacts of these messages under the assumption of “parallel trends” [45]. This assumption means that, in the absence of the intervention, the difference in outcomes (here, appointment attendance and benefit redemption rates) between exposed and non-exposed groups (i.e., participants who received the text and participants who did not) would have not changed over time. Thus, any changes in the magnitude of this difference in outcomes across different intervention phases can be attributed to the intervention itself, even in the case that the exposed and non-exposed groups are dissimilar from one another, as long as such differences are approximately stable over time.

#### 2.6.1. Appointment Attendance Analysis

Logistic regression models assessed the impacts of differently framed appointment reminder texts on appointment attendance using a DiD approach. The main predictors were the intervention phase coded as a two-category predictor variable for each comparison (e.g., baseline vs. gain-framed phase), the exposure group (exposed to text vs. not), and their interaction, which indicated whether the difference in appointment attendance between two exposure groups was different across the two intervention phases under investigation. Specifically, three logistic regression models, one for each comparison (i.e., baseline vs. gain-framed, baseline vs. loss-framed, and gain-framed vs. loss-framed), were run.

The margins command in Stata statistical software version 16 (StataCorp LLC, College Station, TX, USA) estimated predicted appointment attendance rates for each exposure group at each compared intervention phase, while keeping all other predictors at their means. Postestimation commands (i.e., lincom in Stata) assessed whether the differences in predicted attendance rates between the two exposure groups were significantly different across the compared intervention phases.

Models included two-way clustering to account for both the repeated observations of participants over the evaluated period and multiple participants within WIC households using Stata’s vce2way option [46].

#### 2.6.2. Monthly Food Benefits Redemption Analysis

Linear regression models assessed the impacts of differently framed food benefits reminder texts on the proportion of monthly food benefits redeemed using a DiD approach. As in appointment attendance regression models, the main predictors were the binary variables indicating the intervention phase and the exposure group, as well as their interaction terms, which captured whether the difference in outcomes between the exposure groups was different at the two intervention phases under investigation. These linear regression models also included a cluster command to account for repeated observations of WIC households over the compared phases (i.e., vce(cluster) in Stata).

Sensitivity analysis models to assess the CVB redemption outcome were identical to that for overall food benefits redemption, other than that the outcome assessed was the proportion of CVBs redeemed.

## 3. Results

### 3.1. Sample Characteristics

Key characteristics of the sample data from a snapshot month of September 2022, the first month in the intervention period, are displayed in Table 2. The sub-sample displayed in this table included only participants who had at least one scheduled appointment that month. Over half reported also participating in SNAP (54.6%), and most attended a clinic on tribal land (81.6%).

### 3.2. Evaluation Results

#### 3.2.1. Appointment Attendance Results

**Figure 3:**
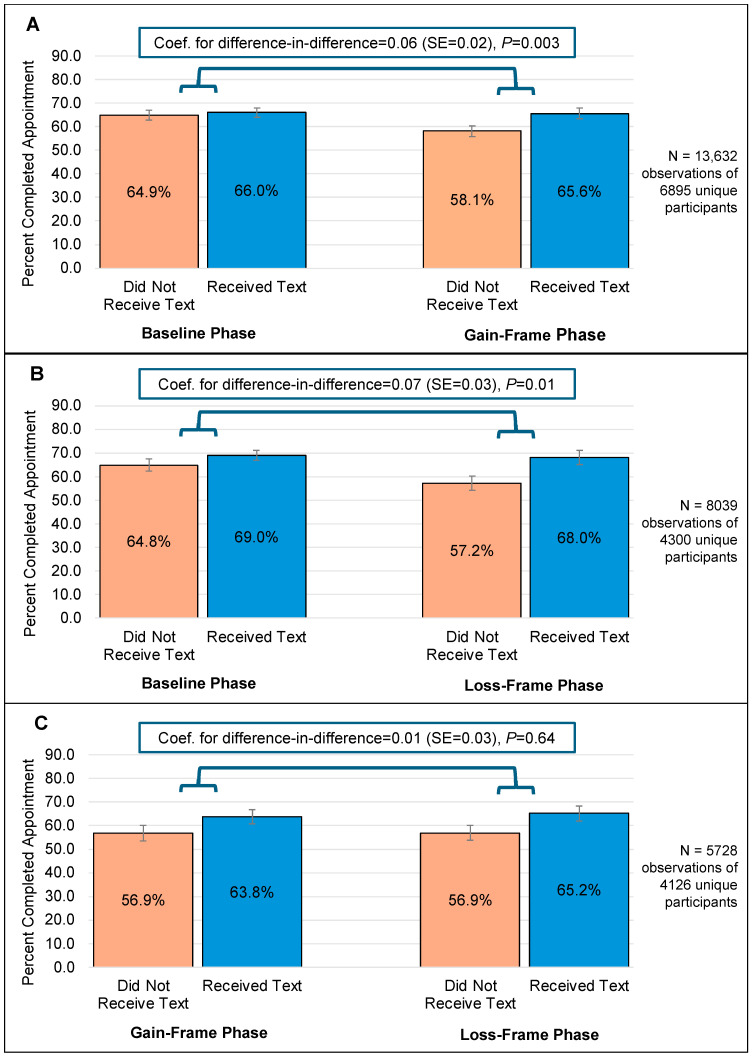
Results from regression models using a difference-in-difference approach to compare appointment attendance rates between reminder text message receipt and non-receipt groups across study phases: Panel (**A**) (baseline vs. gain-framed), Panel (**B**) (baseline vs. loss-framed), and Panel (**C**) (gain-framed vs. loss-framed) among Inter Tribal Council of Arizona WIC participants.

Figure 3 displays appointment attendance rates by exposure group and intervention phase predicted by regression models. The DiD analysis revealed that the change in appointment attendance rates between the baseline and the gain-framed phase was significantly different between the exposed and the non-exposed groups (*p* = 0.003). Specifically, compared to baseline, attendance declined during the gain-framed phase among the non-exposed group, while it remained stable among the exposed group.

Similarly, the DiD analysis showed that the change in appointment attendance rates between the baseline and loss-framed phase was different (*p* = 0.01) between the exposed and the non-exposed groups. As was detected in the baseline vs. gain-framed phase comparison, attendance declined during the loss-framed phase among the non-exposed group, while it remained approximately stable among the exposed group.

The DiD analysis did not detect any differences in appointment attendance rates between exposure groups between the loss-framed and gain-framed phases (*p* = 0.64).

#### 3.2.2. Monthly Food Benefits Redemption Results

**Figure 4:**
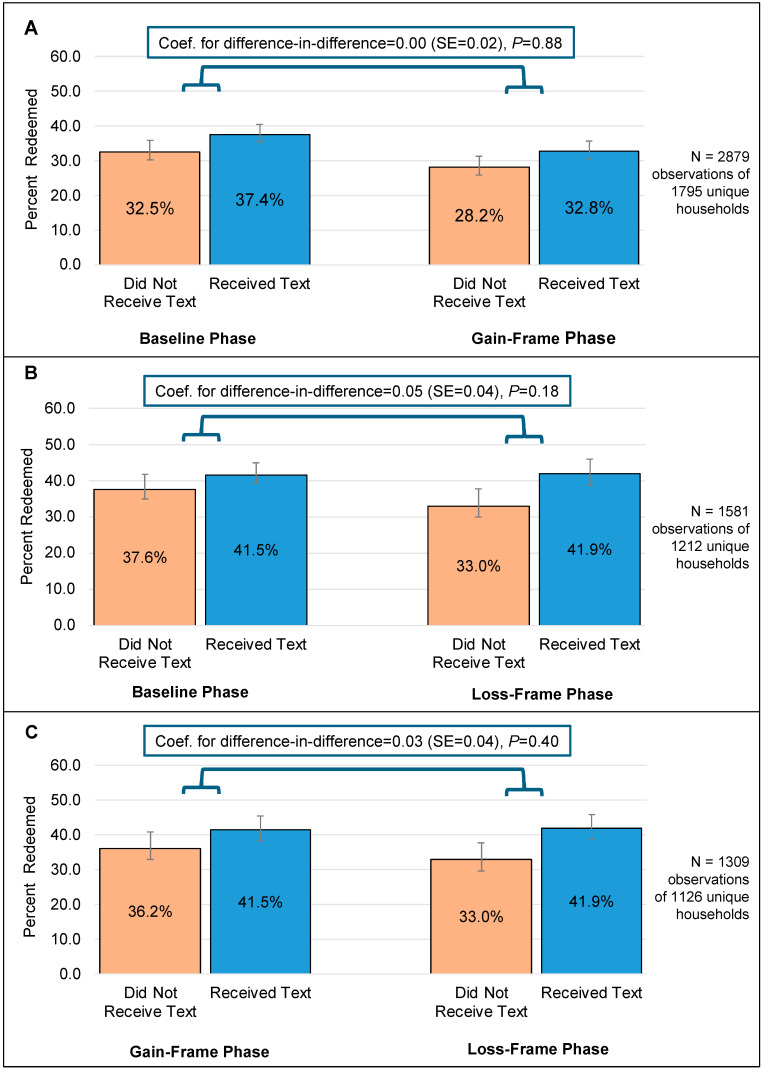
Results from regression models using a difference-in-difference approach to compare household food benefit redemption rates between reminder text message receipt and non-receipt groups across study phases: Panel (**A**) (baseline vs. gain-framed), Panel (**B**) (baseline vs. loss-framed), and Panel (**C**) (gain-framed vs. loss-framed), among Inter Tribal Council of Arizona WIC households.

Differences in redemption rates for the overall monthly food benefits package by treatment group and intervention phase are shown in Figure 4. Unlike for appointment attendance, the DiD analysis did not detect differences in how the food benefits redemption rates changed across intervention phases for exposed vs. non-exposed participants. Patterns of results from sensitivity analyses assessing CVB redemption rates were similar to those for overall food benefits package redemption and are shown in Supplementary Materials Figure S2.

## 4. Discussion

Findings from our evaluation indicated that text message appointment reminders incorporating health communication theory-based message framing improved appointment attendance rates, when compared to standard information-based text messages, among WIC participants within an Indian Tribal Organization WIC state agency. Specifically, appointment attendance rates remained steady for participants who received health communication theory-based appointment reminder messages (either gain-framed or loss-framed), while attendance rates dropped among participants who did not receive the appointment reminders during the gain-framed and loss-framed intervention phases compared to the baseline phase. If the intervention had not occurred, we would have expected similar trends between the exposed and the non-exposed groups. Instead, we observed differing trends, suggesting that the theory-based reminders prevented the appointment attendance rate from dropping for the exposed group as well.

This finding aligns with the study hypotheses. Both the gain-framed and loss-framed text messages resulted in improved appointment attendance compared to the standard information-based text. Improving engagement within WIC is an important goal for many organizations, including the U.S. Department of Agriculture (USDA) Food and Nutrition Service that administers the program [47], the Food Research and Action Center [48], and the National WIC Association [49]. The finding that incorporating health communication theory-based language into messages improved appointment attendance is consistent with previous research conducted in healthcare settings, which also showed that reminder messages containing persuasive language improved appointment attendance when compared to solely information-based messages [26,27,50]. Unlike one previous study conducted in a specialty mental healthcare setting that found gain-framed messages were more effective than loss-framed messages in improving appointment attendance [48], our additional comparisons revealed no difference in effectiveness between the two framing strategies—both were equally effective. To the authors’ knowledge, this is the first study to examine potential impacts of such texts among WIC recipients. Findings from this evaluation indicated that incorporating persuasive language based on health communication principles into appointment reminder messages may be an effective, low-cost strategy that contributes toward achieving this goal of improved WIC participant engagement.

Unlike findings related to appointment attendance, analyses examining impacts of food benefits reminders incorporating persuasive message framing did not detect improvements in food benefits redemption when compared to the originally used text messages. A few reasons could have contributed to the lack of significant findings. First, access to nearby WIC-authorized retailers, such as supermarkets, where families can redeem their food benefits influences redemption [18]. American Indians in particular face significant barriers to accessing healthy foods due to inequitable food systems [51,52]. These challenges are particularly pronounced on tribal lands, where access to fresh and affordable fruits and vegetables is consistently lower than in other parts of the U.S. [10,53]. Only 26% of individuals living on tribal lands reside within walking distance to a supermarket, compared with 59% of all individuals in the U.S. Additionally, supermarkets on tribal lands often have a limited selection of healthy food options, including fresh fruits and vegetables in good condition [54]. High poverty rates on tribal lands further exacerbate these accessibility barriers [55]. Since most ITCA WIC participants live on tribal lands, they may experience heightened challenges to redeeming their benefits due to these compounded systemic barriers. Addressing these access-related challenges to benefit redemption likely requires more persuasive messaging alone. Indeed, redemption of food benefits was relatively low during the study period, with households in the analytical sample redeeming less than one-third of their available benefits. Another possible contributor to these low redemption rates is that ITCA WIC was batch issuing food benefits, as allowed by USDA waivers issued during the COVID-19 pandemic [56], throughout the study period. This means that all participants were eligible to receive food benefits each month, regardless of whether they attended their appointments. Therefore, food benefits reminder texts could also have been received by participants who were not actively engaged in the program and who may not have been aware that they had benefits to use. While not the focus of the DiD analysis, it is of note that households that received a food benefits reminder text message redeemed a higher proportion of their monthly food benefit package compared to those that did not during each of the three phases, indicating that text messages may be an effective strategy for WIC agencies to increase benefit redemption. Finally, the appointment reminders previously sent by the ITCA WIC serving as the comparison standard message solely included information about upcoming appointments. On the other hand, standard text messages serving as the comparison message for food benefits reminders included some persuasive language (i.e., “…WIC is here to help you feed your family. Don’t forget to buy your healthy WIC foods before {Date}”).

It is also notable that a relatively high proportion (nearly 20%) of attempted text messages were not received by participants due to message failure. Unreliable cell coverage is particularly common on tribal lands [57]. As such, expanding cellular provider coverage in underserved areas would increase the reach of persuasive text messages among WIC participants living on tribal lands.

### Limitations

There are some limitations to this evaluation. First, due to constraints of the technological platform used to send the texts, it was not possible to conduct a randomized evaluation in which participants could be randomly assigned to receive one of the three text types. Instead, we used a DiD approach to compare differences between exposure groups (text received vs. text not received) across study phases and controlled for participant demographics that could have differed across phases and text reception status, such as race, ethnicity, household WIC beneficiary composition, and household SNAP participation status. While this approach helped mitigate potential selection bias and unobserved heterogeneity, it could not fully eliminate biases arising from unmeasured confounders. Specifically, DiD may not account for unmeasured confounders that (i) change over time at a different rate between the two groups compared, and (ii) have a time-varying association with the outcome. Nonetheless, future studies assessing impacts of theory-based messages on program engagement would benefit from using a randomized design to better account for both observed and unobserved factors, such as cellular phone access and coverage, that may differ between WIC-participating households. Second, while unique messages were developed for breastfeeding-specific appointments and nutrition-education-specific appointments, the analyses could not be stratified by the type of message received due to the relatively small sample sizes of participants who received those specific texts. Finally, this intervention took place within a WIC state agency serving a predominantly American Indian population; therefore, findings may not be generalizable to other WIC populations with different demographic or geographic characteristics.

## 5. Conclusions

WIC provides important nutritional support for families with young children at risk of food insecurity and advances equity; however, participation in WIC does not keep up with the high rates of food insecurity, and this is particularly the case within American Indian communities. This evaluation showed that incorporating health communication theory-based message framing into appointment reminder texts improved WIC appointment attendance rates above and beyond standard information-based texts among participants served by an Indian Tribal Organization WIC state agency. Findings suggested that persuasive text message reminders based on health communication principles may be an effective, low-cost strategy to improve appointment attendance within the WIC program among eligible American Indian families. On the other hand, message framing did not result in improved benefit redemption rates. To improve benefit redemption, broader, multifaceted strategies, such as increasing access to WIC-authorized food retailers and addressing transportation barriers, may be necessary.

## Figures and Tables

**Table 1:** Message framing of the 16 reminder text messages developed by the research team to be considered during the pretesting phase through key informant interviews with 15 Inter Tribal Council of Arizona WIC local agency staff members.

Message Reminder Type	Message Frame	
	Gain Frame	Loss Frame
General Appointment Reminder	Social norm ^a^ Health focus ^b^ Financial focus ^c^	Social norm Health focus Financial focus
Food Benefits Reminder	Social norm Health focus Financial focus	Social norm Health focus Financial focus
Nutrition Education Appointment Reminder ^d^	Health focus	Health focus
Breastfeeding Appointment Reminder	Both health and financial focus	Both health and financial focus

^a^ Social norm messages included statistics on the number of families WIC serves. ^b^ Health focus messages mentioned that WIC helps families be healthy. ^c^ Financial focus messages highlighted how WIC can help save families money. ^d^ Because nutrition is an inherently health-focused topic, nutrition education appointment reminders were adapted to be health-focused only.

**Table 2:** Characteristics of Inter Tribal Council of Arizona WIC participants who had at least one appointment scheduled during September 2022, the first evaluated month of the text messaging intervention (n = 3474).

Sample Characteristics	Mean ± SD or n (%)
Participating in SNAP, (n) %	1897 (54.6)
Participant type, (n) %	
Pregnant woman	305 (8.8)
Postpartum/breastfeeding woman	383 (11.0)
Infant	663 (19.1)
Child	2123 (61.1)
Participant age in years, Mean ± SD	
Pregnant woman	27.0 ± 6.0
Postpartum/breastfeeding woman	27.2 ± 5.9
Child	2.4 ± 1.2
Infant (age in months)	5.2 ± 3.3
Number of WIC participants in household, Mean ± SD	1.88 ± 0.9
Attending clinic on tribal land, % (n) %	2833 (81.6)

## Data Availability

The datasets generated and/or analyzed during the current study are not readily available because the data belong to a tribal entity.

## Supplementary Materials

The following supporting information can be downloaded at: https://www.mdpi.com/article/10.3390/nu17071112/s1. Table S1: Text messages sent to Inter Tribal Council of Arizona WIC participants during the text messaging intervention. Figure S1: Description of the analytical samples for comparisons assessing impacts of theory-based text messages on household cash value benefit (CVB) for fruits and vegetables redemption rates among Inter Tribal Council of Arizona WIC-participating households. Figure S2: Results from regression models using a difference-in-difference analytical approach to compare differences in household cash value benefit (CVB) for fruits and vegetables redemption rates between text message receipt and non-receipt groups across compared study phases among Inter Tribal Council of Arizona WIC-participating households.

## Abbreviations

The following abbreviations were used in this manuscript:

CVB	Cash value benefit
DiD	Difference-in-difference
ITCA	Inter Tribal Council of Arizona
SNAP	Supplemental Nutrition Assistance Program
WIC	Special Supplemental Nutrition Program for Women, Infants, and Children
