# Supplementary material for: Health Communications Theory-Based Text Message Reminders Boost Special Supplemental Nutrition Program for Women, Infants, and Children (WIC) Appointment Attendance Among American Indian Populations
Source: Nutrients. 2025 Mar 22;17(7):1112. doi: 10.3390/nu17071112 (PMC11990218; doi:10.3390/nu17071112)
Supplement: Supplementary file 1 [file nutrients-17-01112-s001.zip › nutrients-3527299-supplementary.pdf]

SUPPLEMENTARY MATERIAL

Table S1. Text messages sent to Inter Tribal Council of Arizona WIC participants during the text messaging intervention.

Message Type	Message
General Appointment Reminder	
Standard message	This is {clinic_name} with a reminder that you have an appointment on {appointment [appt] date} at {appt_time}. Text C to confirm your appointment. Text R to reschedule.
Gain-frame message	Hi {Caregiver Name}, it's WIC! Your appointment is on {appt_date} at {appt_time}. You work hard to keep your family healthy – WIC is here to help! WIC saves you money on healthy food. Text C to confirm your appointment. Need to reschedule? Text R to reschedule.
Loss-frame message	Hi {Caregiver Name}, it's WIC! Your appointment is on {appt_date} at {appt_time}. You work hard to keep your family healthy – WIC is here to help! Don't miss out on healthy WIC foods. Text C to confirm your appointment. Need to reschedule? Text R to reschedule.
Food Benefits Reminder	
Standard message	{Caregiver Name}, WIC is here to help you feed your family. Don't forget to buy your healthy WIC foods before {Date}.
Gain-frame message	Hi {Caregiver Name}, it's WIC! Your WIC benefits will expire on {Date}. Remember, you get WIC dollars to buy fruits & vegetables of your choice! WIC foods help your family be healthy & strong.
Loss-frame message	Hi {Caregiver Name}, it's WIC! Your WIC benefits will expire on {Date}. Don't miss out on buying fruits & vegetables of your choice with WIC dollars! WIC foods help your family be healthy & strong.
Breastfeeding Specific Appointment Reminder	
Standard message	This is {clinic_name} with a reminder that you have an appointment on {appt_date} at {appt_time}. Text C to confirm your appointment. Text R to reschedule.
Gain-frame message	Hi {Caregiver Name}, it's WIC! Your appointment with our breastfeeding expert is on {appt_date} at {appt_time}. Did you know breastfeeding mothers receive extra food benefits? Breast milk is the best food for your baby & WIC is here to support you. Text C to confirm your appointment. Need to reschedule? Text R to reschedule.
Loss-frame message	Hi {Caregiver Name}, it's WIC! Your appointment with our breastfeeding expert is on {appt_date} at {appt_time}. Did you know breastfeeding mothers receive extra food benefits? Breast milk is the best food for your baby. Don't miss out on breastfeeding support from WIC. Text C to confirm your appointment. Need to reschedule? Text R to reschedule.
Nutrition Education Specific Appointment Reminder	
Standard message	This is {clinic_name} with a reminder that you have an appointment on {apt_date} at {apt_time}. Text C to confirm your appointment. Text R to reschedule.

Gain-frame message	Hi {Caregiver Name}, it's WIC! Your nutrition education appointment is on {apt_date} at {apt_time}. // Our nutrition tips help you make the most of WIC. Text C to confirm your appointment. Need to reschedule? Text R to reschedule.
Loss-frame message	Hi {Caregiver Name}, it's WIC! Your nutrition education appointment is on {apt_date} at {apt_time}. // Don't miss out on nutrition tips to help you make the most of WIC. Text C to confirm your appointment. Need to reschedule? Text R to reschedule.

Figure S1. Description of the Analytical Samples for Comparisons Assessing Impacts of Theory-Based Text Messages on Household Cash-Value Benefit for Fruits and Vegetables (CVBs) Redemption Rates Among Inter Tribal Council of Arizona WIC Participating Households. [a] Excluded participants to eliminate possibility of learning effect from gain-framed theory-based messages. [b] Includes participant observations for which recipients did not receive text due to (a) previously opting out of receiving text messages, (b) message delivery failure, (c) not providing a valid cell phone number, or (d) scheduling an appointment less than two days in advance

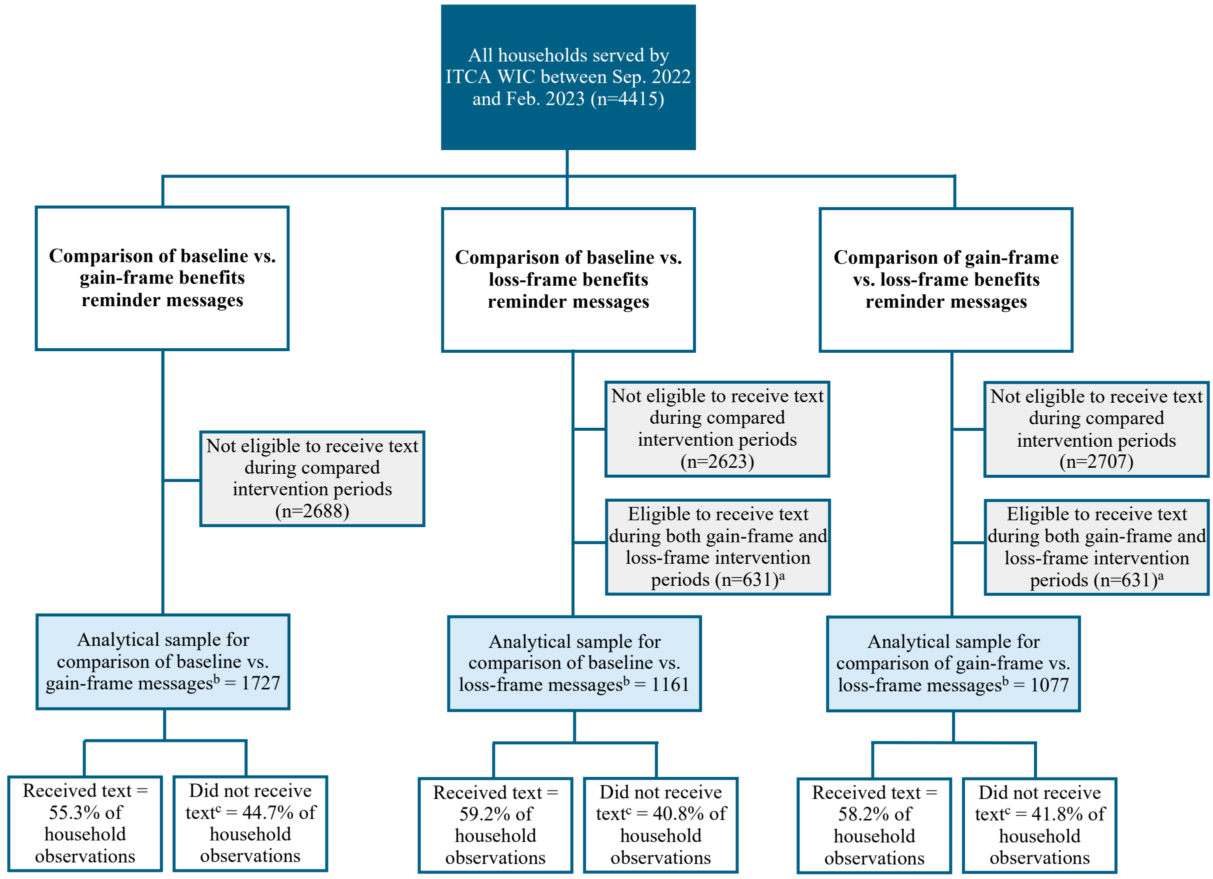

Figure S2. Results From Regression Models Using a Difference-in-Difference Analytical Approach to Compare Differences in Household Cash-Value Benefit for Fruits and Vegetables (CVB) Redemption Rates Between Text Message Receipt and Non-receipt Groups Across Compared Study Phases Among Inter Tribal Council of Arizona WIC Participating Households.

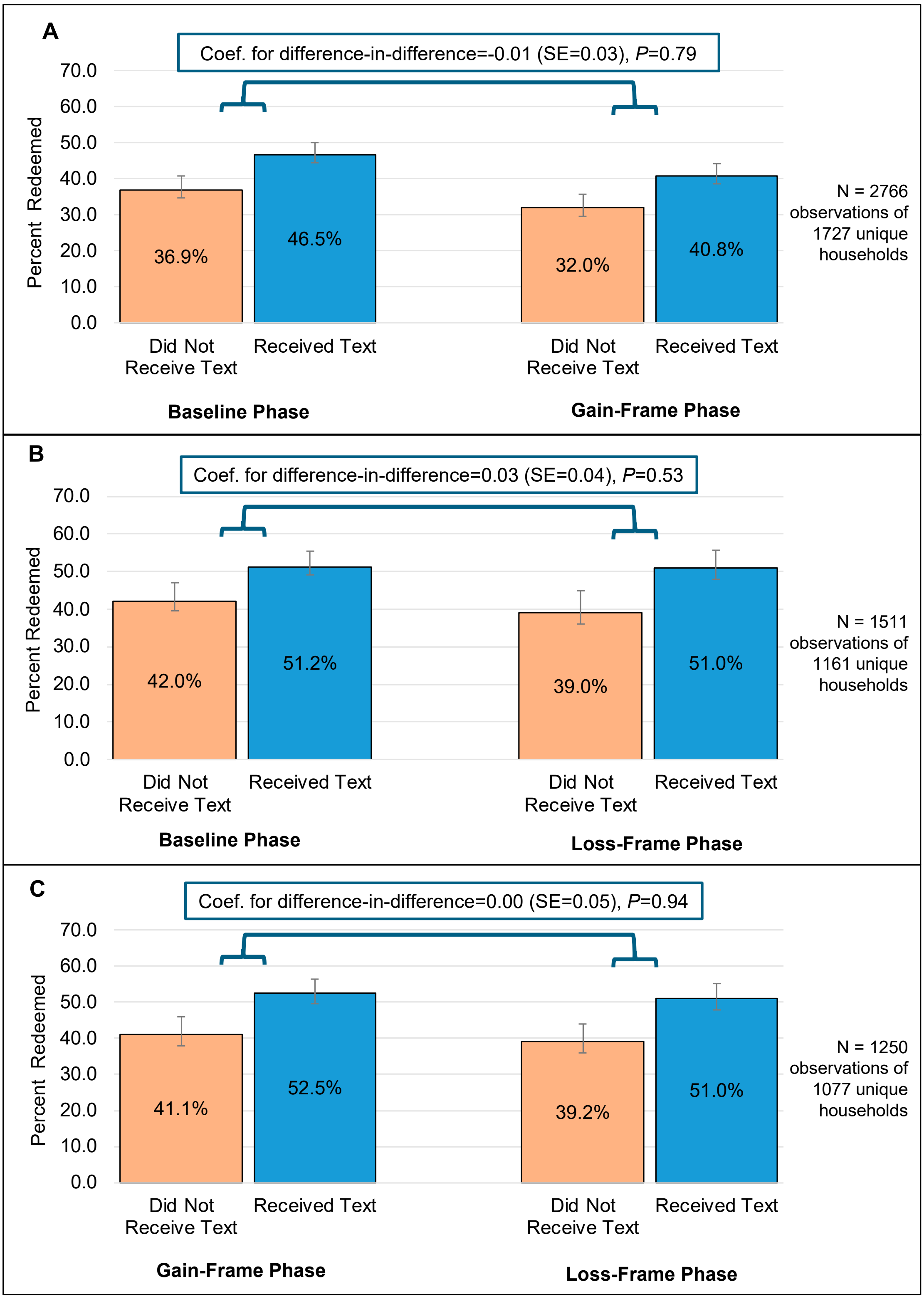
A
Coef. for difference-in-difference=-0.01 (SE=0.03), P=0.79
Percent Redeemed
70.0
60.0
50.0
40.0
30.0
20.0
10.0
0.0
36.9%
46.5%
32.0%
40.8%
Did Not Receive Text
Received Text
Did Not Receive Text
Received Text
Baseline Phase
Gain-Frame Phase
N = 2766 observations of 1727 unique households
B
Coef. for difference-in-difference=0.03 (SE=0.04), P=0.53
Percent Redeemed
70.0
60.0
50.0
40.0
30.0
20.0
10.0
0.0
42.0%
51.2%
39.0%
51.0%
Did Not Receive Text
Received Text
Did Not Receive Text
Received Text
Baseline Phase
Loss-Frame Phase
N = 1511 observations of 1161 unique households
C
Coef. for difference-in-difference=0.00 (SE=0.05), P=0.94
Percent Redeemed
70.0
60.0
50.0
40.0
30.0
20.0
10.0
0.0
41.1%
52.5%
39.2%
51.0%
Did Not Receive Text
Received Text
Did Not Receive Text
Received Text
Gain-Frame Phase
Loss-Frame Phase
N = 1250 observations of 1077 unique households
